# Synthesis and Characterization of β-Cyclodextrin Functionalized Ionic Liquid Polymer as a Macroporous Material for the Removal of Phenols and As(V)

**DOI:** 10.3390/ijms15010100

**Published:** 2013-12-23

**Authors:** Muggundha Raoov, Sharifah Mohamad, Mhd Radzi Abas

**Affiliations:** 1 University of Malaya Centre for Ionic Liquids, Department of Chemistry, Faculty of Science, University of Malaya, Kuala Lumpur 50603, Malaysia; E-Mail: muggundha_raoov@hotmail.com; 2 Department of Chemistry, Faculty of Science, University of Malaya, Kuala Lumpur 50603, Malaysia; E-Mail: radzi@um.edu.my; 3 Advanced Medical & Dental Institute, University of Science Malaysia, No 1–8 (Lot 8), Persiaran Seksyen 4/1, Bandar Putra Bertam, Kepala Batas, Pulau Pinang 13200, Malaysia

**Keywords:** ionic liquid, Cyclodextrin, polymer, adsorption

## Abstract

β-Cyclodextrin-ionic liquid polymer (CD-ILP) was first synthesized by functionalized β-cyclodextrin (CD) with 1-benzylimidazole (BIM) to form monofunctionalized CD (βCD-BIMOTs) and was further polymerized using a toluene diisocyanate (TDI) linker to form insoluble CD-ILP (βCD-BIMOTs-TDI). The βCD-BIMOTs-TDI polymer was characterized using various tools and the results obtained were compared with those derived from the native β-cyclodextrin polymer (βCD-TDI). The SEM result shows that the presence of ionic liquid (IL) increases the pore size, while the thermo gravimetric analysis (TGA) result shows that the presence of IL increases the stability of the polymer. Meanwhile, Brunauer-Emmett-Teller (BET) results show that βCD-BIMOTs-TDI polymer has 1.254 m^2^/g surface areas and the Barret-Joyner-Halenda (BJH) pore size distribution result reveals that the polymer exhibits macropores with a pore size of 77.66 nm. Preliminary sorption experiments were carried out and the βCD-BIMOTs-TDI polymer shows enhanced sorption capacity and high removal towards phenols and As(V).

## 1. Introduction

Cyclodextrin (CDs) are a well-known series of macro cyclic oligosaccharides resulting from the degradation of starch by bacterial enzymes. Generally, CDs are composed of 6, 7, or 8 D-glucose units connected by α-1,4-glucosidic linkages which are categorized as α-, β- and γ-CD, respectively. Every D-glucose unit consists of three free hydroxyl units groups, which differ in their reactivity and functions [1]. The entire primary hydroxyl group at the 6-positions of the D-glucose units is on the opposite side of the ring while the entire secondary hydroxyl group at the 2- and 3-positions is on one side of the torus [2]. The most notable feature of β-CD is its ability to form solid inclusion compounds (host-guest complexes) with a very wide range of solid, liquid, and gaseous compounds by molecular complexation [3–5] and through various kinds of interaction (van der waals force, hydrophobic interaction, electrostatic affinity, dipole-dipole interaction, and hydrogen bonding) [6]. Due to specific properties of CDs polymers, they are able to cater for a wide range of research and application [7]. Examples of applications include chemical separations [8], adsorbents [9,10] food processing [11] and pharmaceutical excipients [12].

Ionic liquid (ILs), is defined as a kind of salt in which the ions are poorly coordinated. Consequently these compounds are liquid below 100 °C or even at room temperature (RTIL’s) [13]. They have several unique properties namely non-volatility, non-flammability, low viscosity, chemical and electrochemical stability [14] and remain in the liquid state over a wide temperature range. ILs can also be designed to be environmentally benign, with large potential benefit for sustainable chemistry [15]. ILs are considered as template solvents in some syntheses due to its ability to self-assemble in different domains (polar and nonpolar) and these properties of ILs have been transferred to the development of supramolecular polymers [16]. However, a novel class of IL polymers has become famous among researchers since these materials exhibit the properties of ILs and the polymers [17]. Owing to the properties of β-CD polymers and ILs, the functionalization of β-CD with the IL has attracted our interest with a view to prepare a new generation of macroporous materials which may demonstrate interesting phenomena in adsorption studies.

Previously, CD-IL materials were widely applied in separation sciences due to their importance as well as being intriguing [18,19]. In contrast, application of the CD-ILP in the removal of toxic pollutants is still in its early stages; thus academic interest in combinations of ILs and CD is increasing. In this study β-CD is functionalized with the IL and further polymerized with an isocyanate linker to form an insoluble polymer for wider applications. The structure and properties of the βCD-BIMOTs-TDI polymer are studied and compared with native β-CD-TDI polymers. It is found that the βCD-BIMOTs-TDI polymer shows enhanced sorption properties when tested with phenols and As(V).

## 2. Results and Discussion

### 2.1. Preparation of 6-*O*-Monotosyl-6-deoxy-β-cyclodextrin (β-CDOTs) (1) and Structural Characterization by ^1^H NMR

**Figure 1.**
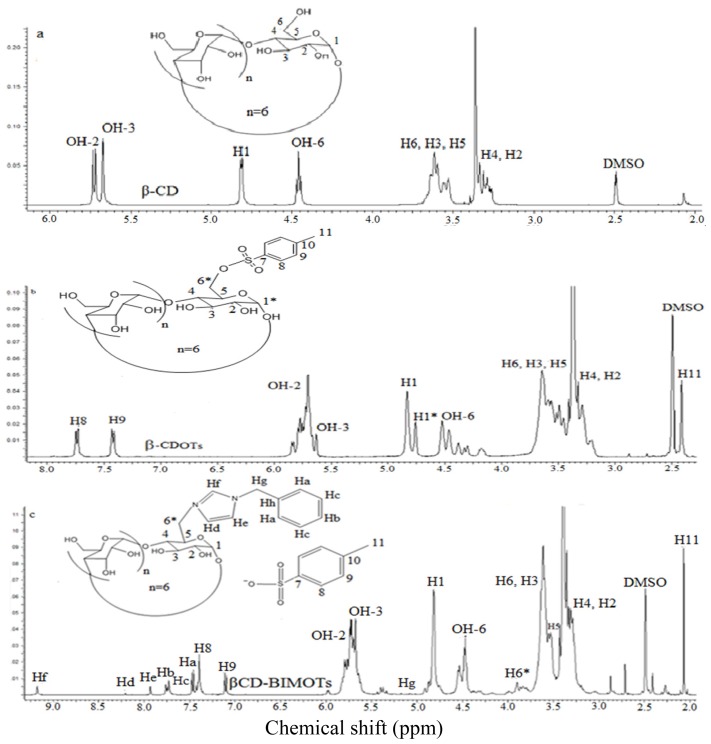
NMR spectrum of (**a**) β-cyclodextrin (βCD); (**b**) 6-*O*-Monotosyl-6-deoxy-β-cyclodextrin (βCD-OTs), and (**c**) Mono-6-deoxy-6-(3-benzylimidazolium)-β-cyclodextrin (βCD-BIMOTs).

β-CDOTs is the most important intermediate product in order to further functionalize the primary hydroxyl groups (C_6_) of β-CD, since tosylate is a good leaving group and can be easily substituted by other nucleophiles. The reaction has been carried out with the presence of a base (NaOH), where the proton of C_6_ will be removed in order to make O^−^ which is a nucleophile that can be easily substituted, so when a CD reacts with the tosyl group in a basic medium, the monotosylation at C_6_ (β-CDOTs) is produced. Various methods have been reported previously to prepare β-CDOTs under different conditions [20,21] but, as there are some limitations in these methods, in this study, *p*-toluene sulfonic anhydride (Ts_2_O) is used instead of TsCl [22]. This method can be used in large-scale synthesis as it is easy to carry out and can produce a high percentage of yield. The DT (%) is found to be less than 1, further suggesting that tosylation at primary hydroxyl of β-CD has successfully occurred. The formation of β-CDOTs is confirmed using various tools and the results are summarized in the experimental section. The result shows that β-CDOTs have been successfully prepared. A new peak doublet of the doublet proton signal appeared around 4.8 ppm, and belongs to the H_1_* proton (Figure 1b) of a substituted CD and Figure 1a shows ^1^H NMR spectrum of β-CD.

### 2.2. Preparation of Mono-6-deoxy-6-(3-benzylimidazolium)-β-cyclodextrin (βCD-BIMOTs) (2) and Structural Characterization by ^1^H NMR

Figure 1c shows the ^1^H NMR spectrum of compound **2** in the d_6_-DMSO solvent. The product formed was found to dissolve in water and several organic solvents (DMF, DMS0 and ethanol). The formation of βCD-BIMOTs was further confirmed on the use of various tools. The entire proton is assigned well and shown clearly in Figure 1c. Protons of imidazole ring (H_f_, H_e_, H_d_) appeared in the downfield region since the protons are de-shielded upon functionalization. New peak is observed in proton (H_6_*, 3.95 ppm) and carbon signal (C_6_*, 42.5 ppm), belongs to the substituted CD.

### 2.3. Preparation of Insoluble β-Cyclodextrin Polymers

The polymerization reactions of the diisocyanate linkers with βCD-BIMOTs and βCD were monitored through IR spectroscopy. The obtained polymers were produced in high yields and found to be insoluble in water as well as organic solvents (e.g., DMSO, D_2_O, DMF, CHCL_3_, CH_3_CN) and this enabled us to use them in water treatment.

### 2.4. FT-IR Analysis

**Figure 2.**
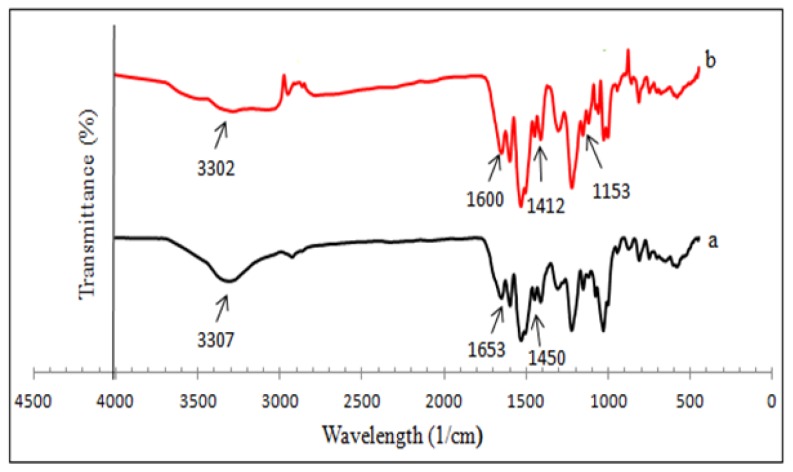
FT-IR analysis of (a) βCD-TDI polymer and (b) βCD-BIMOTs-TDI polymer.

The spectrums of insoluble β-cyclodextrin polymers (βCD-BIMOTs-TDI and βCD-TDI) are shown in Figure 2. The absence of a peak at 2270 cm^−1^ (corresponding to the isocyanate group) and the presence of carbamate linkage NHCO, is clearly observed which indicates the completion of the reaction [23]. Main IR frequencies with assignments are shown in Table 1. The broad –OH stretching band of the β-CD around 3300 cm^−1^ that corresponds with the multiple –OH functional groups, is reduced upon cross-linking with TDI and its width is ascribed to the formation of inter and intramolecular hydrogen bonds. In the FT-IR spectrum of βCD-BIMOTs-TDI polymer, the band at 1153 cm^−1^ is attributed to the imidazolium groups, which further supports the anchoring of the ILs molecule onto the cyclodextrin surface. Therefore, we conclude that the polymerization between βCD-BIMOTs, βCD and TDI has been completed and the polymers (βCD-BIMOTs-TDI, βCD-TDI) are formed.

### 2.5. X-ray Diffraction (XRD) Analysis

**Figure 3.**
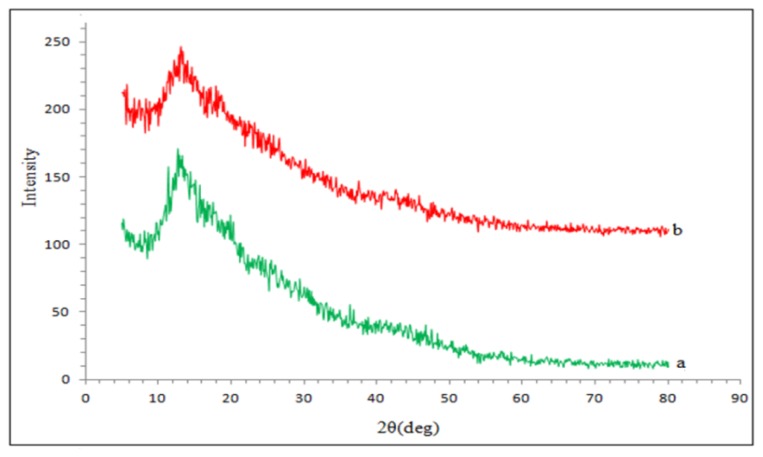
XRD analysis of (a) βCD-TDI and (b) βCD-BIMOTs-TDI.

Further evidence for the formation of the CD-ILP (βCD-BIMOTs-TDI) was obtained through the X-ray powder diffraction (XRD) as demonstrated in Figure 3. Basically, no peaks are clearly visible in the XRD of the polymers, which is due to the change of cystalloids after crosslinking with TDI. It was found that the peak at 2θ = 13° obviously decreases and broadens in βCD-BIMOTs-TDI polymer which indicates that the degree of crystallinity decreases more as compared to the unmodified βCD-TDI polymer [24]. The lack of crystalline in the polymers may be due to the loss of regularity throughout the polymeric chains, which in turn, is due to the introduction of bulky TDI molecules. In this study, it is confirmed that βCD-TDI and βCD-BIMOTs-TDI polymers are amorphous.

### 2.6. Surface Morphology Studies

**Figure 4.**
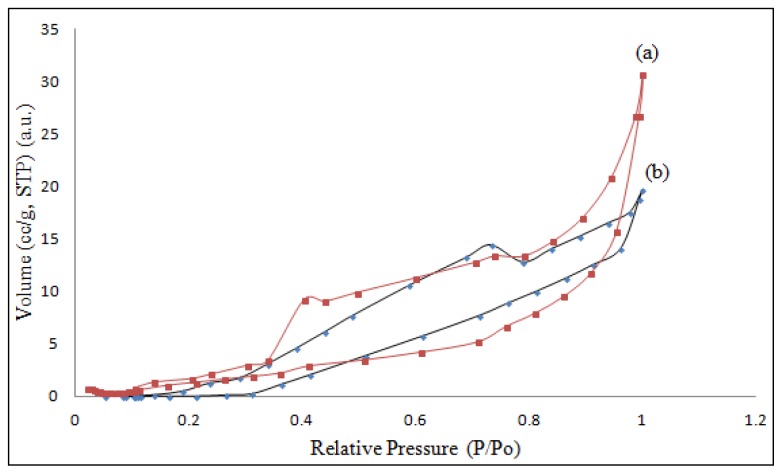
Nitrogen adsorption-desorption isotherms of (a) βCD-BIMOTs-TDI and (b) βCD-TDI.

The N_2_ adsorption/desorption isotherm (Figure 4) for βCD-BIMOTs-TDI polymer exhibits a typical type-II isotherm with H_3_ type hysteresis loop according to the IUPAC [25], which indicates that the macroporous structure, with good pore connectivity, might be present in polymer compared to βCD-TDI polymer which exhibits a typical type-IV isotherm with a steep desorption branch and H_3_ type hysteresis loop. Furthermore, the nitrogen amount adsorbed by βCD-BIMOTs-TDI polymer increases very steeply at high relative pressure (*P*/*P*_0_ > 0.85) which indicates the presence of the macropores [26–28] and the results agree well with the IUPAC definition, which classifies the adsorbent pore into three: micropores (diameter < 2 nm), mesopores (2–50 nm) and macropores (>50 nm). Based on the BJH pore size distribution (calculated from Barret-Joyner-Halenda model) for dry βCD-BIMOTs-TDI polymer exhibited βCD-BIMOTs-TDI polymer was a macroporous material with a pore size of 77.6 nm and pore volume of 0.02435 cm^3^/g. Meanwhile, βCD-TDI polymer represents micropores with pore size of 1.585 nm and pore volume of 0.02188 cm^3^/g. The removal of solid particles which are embedded on the surface of polymers by washing or etching can lead to the formation of pores. The presence of the macropores could be derived from the self-porogen effect during the polymerization process [29].

Furthermore, βCD-BIMOTs-TDI polymers exhibit low specific BET surface area (1.254 m^2^/g) (dry state) after the chemical modification with IL, compared to the native βCD-TDI (2.401 m^2^/g) polymer. Similar results have been obtained in the preparation of macroporous IL material which exhibits low surface area [30]. This phenomenon could be correlated with the covering of the adsorption sites by organic moieties immobilized on the mineral surface (cyclodextrin), which further hinders the N_2_ molecules access to the binding site [31]. Furthermore, βCD-BIMOTs-TDI polymer has hydrogel nature with high swelling capacity in water due to many cavities which allows rapid diffusion process for the adsorbates [32]. The surface area and pore size could greatly increase after swelling. The low surface area could also be from the usage of larger cation parts of IL (1-benzylimidazole) [33]. In addition, the nitrogen desorption at 0.4 *P*/*P*_0_ for βCD-BIMOTs-TDI polymer and 0.75 *P*/*P*_0_ for βCD-TDI polymer are higher due to the heterogeneous surface of the polymers with many cavities (cyclodextrin), imidazolium (βCD-BIMOTs-TDI) and isocyanate group which makes desorption of nitrogen gas difficult. Table S1 shows the structural parameters of the samples.

### 2.7. Scanning Electron Microscope (SEM) Analysis

**Figure 5.**
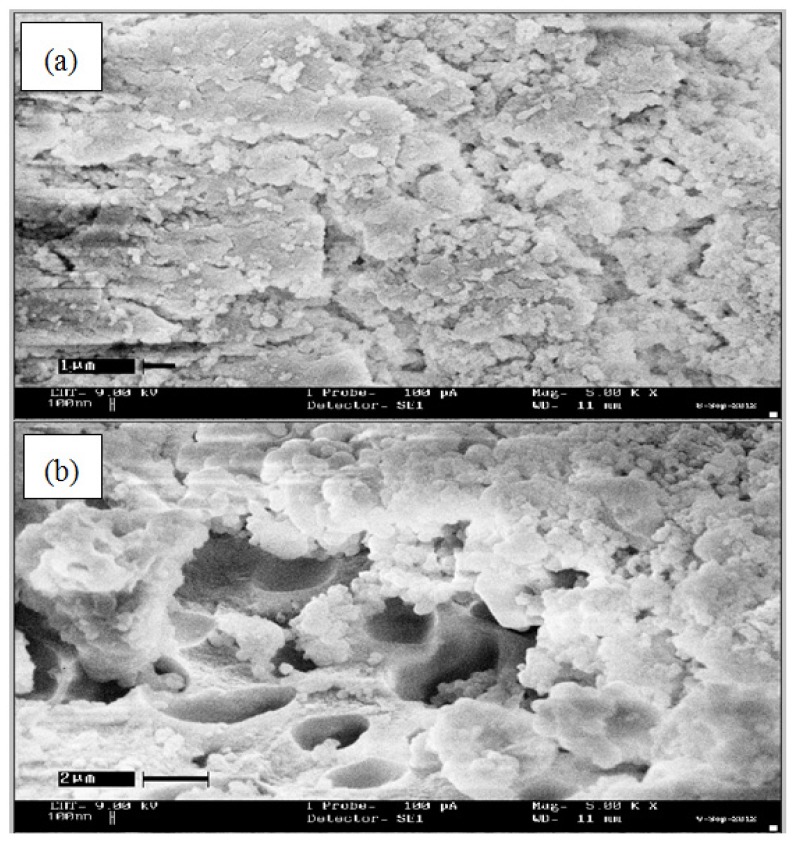
SEM analysis of (**a**) βCD-TDI (**b**) βCD-BIMOTs-TDI (Mag 5.00 KX).

All the microscopic morphological structures were performed using the Scanning Electron Microscope (SEM) in order to determine and compare the surface features of βCD-BIMOTs-TDI polymer with native βCD-TDI polymer. The SEM micrographs of the polymers are shown in Figure 5a,b. From the SEM micrographs, the presence of IL increases the pore size of the βCD-BIMOTs-TDI polymer compared to βCD-TDI polymer and this observation is supported by the BJH result. βCD-TDI polymer reveals a “shrinking” crystal structure. It exhibits the loss of sphericity, smooth surface and reduced size of particles as shown in Figure 5a, while βCD-BIMOTs-TDI polymer exhibits a totally different crystalline structure, which is not comparable with the morphology of the βCD-TDI polymer. Different morphologies are observed for the IL material due to its unique properties. Basically, pore formation depends on the chemical structure of the polymer backbone [34]. In addition, hydrogen-bonding interactions influence the phase separation at a local level during polymerization, which leads to domains that are either polymer-rich or solvent rich. The presence of cyclodextrin, in polymer-rich domains is likely to be highly ordered because of the rigid structure of the cyclodextrin itself. Upon removal of the solvent and completion of the polymerization, micropores remain [34].

### 2.8. Thermal Analysis of the Polymers

#### 2.8.1. Thermo Gravimetric Analyses (TGA)

**Figure 6.**
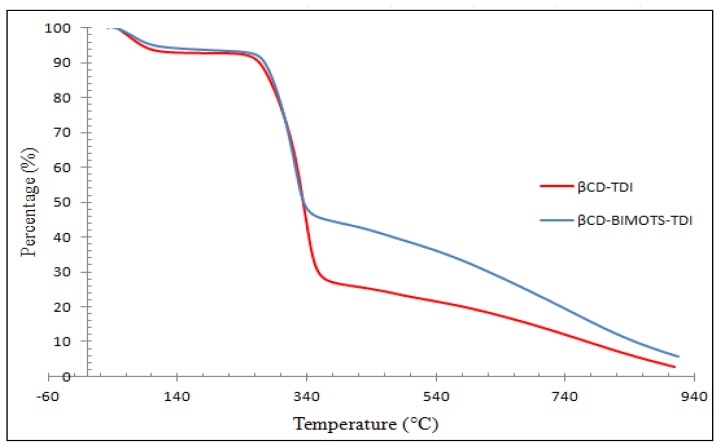
TGA analysis of βCD-BIMOTs-TDI and βCD-TDI.

The TGA curves of βCD-BIMOTs-TDI and βCD-TDI polymers are shown in Figure 6. The thermal behaviors of the polymers (βCD-TDI, βCD-BIMOTs-TDI) involve only a 3-step process. Generally, the first step can be interpreted due to the loss of water; the second and third steps may account for most of the weight and associated with the formation of the residue of the CD polymer. Basically, the second degradation stage of βCD-BIMOTs-TDI polymer takes place at a higher temperature (270–357 °C) with low weight loss (46%), and this result indicates that βCD-BIMOTs-TDI polymer is more stable than the unmodified βCD-TDI polymer which takes place at 260–365 °C with weight loss of 68%. This shows that, the stability of β-CD increases after modification in the polymeric form [35]. Apart from that, high stability of βCD-BIMOTs-TDI polymer could be due to the strong electrostatic interaction between the BIM cation and OTs anion, which enables this material to be used in high-temperature applications. Basically, differences could only be found in the water loss, the onset temperature and the mass loss at a given temperature between modified and unmodified polymers. The degradation and weight loss steps of the polymers are shown in Table 2.

#### 2.8.2. Differential Scanning Calorimetry (DSC)

**Figure 7.**
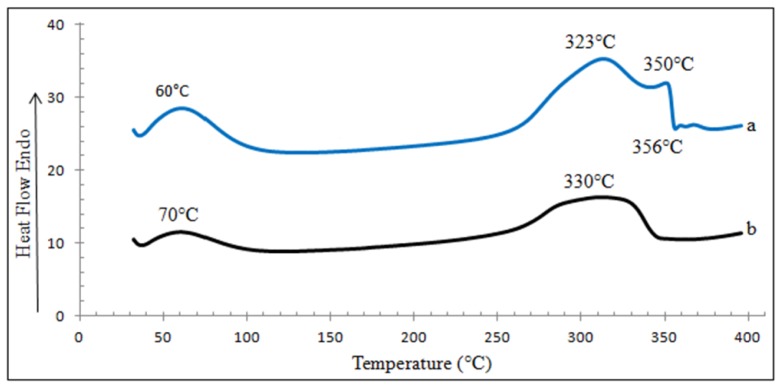
DSC analysis of (a) βCD-TDI and (b) βCD-BIMOTs-TDI.

It can be observed in Figure 7, that the native βCD-TDI polymer, displays different trends compared to βCD-BIMOTs-TDI polymer. βCD-TDI polymer involves four stages in the DSC analysis while βCD-BIMOTs-TDI polymer only involves two stages (Table S2). Endothermic peak had been observed from about −100 °C due to the loss of water. Meanwhile the second endothermic regions between 190 and 350 °C, are associated with the melting ranges of the samples. However, the difference could be observed in the βCD-TDI polymer at around 350 °C where it shows the third endothermic peak due to the melting of the polymer and exothermic peak at 356 °C associated with curing (a process during which a chemical reaction or physical action takes place) of the polymer due to the high degree of cross-linking in the βCD-TDI polymer [34]. The second endothermic peak of βCD-BIMOTs-TDI polymer takes place at a higher temperature (330 °C) compared to βCD-TDI polymer (323 °C). This result shows that the presence of ionic liquid increases the stability of the polymer compared to the native βCD-TDI polymer. This could be due to the properties of ILs that can support the higher temperature range and this IL material is also stable and non-volatile [36].

### 2.9. Sorption Studies

**Figure 8.**
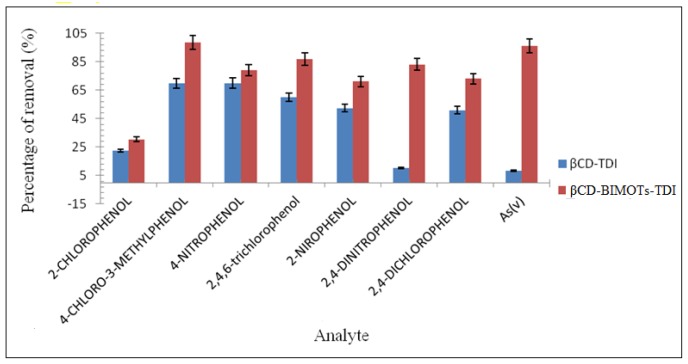
Preliminary batch sorption experiment. (Results based on three replicate analyses for all analytes). Removal condition: 25°C, 10 mL analyte solution (neutral condition), 20 mg sorbent, 180 rpm. Equilibrium time (H): phenols 2H, As(V) 17H.

**Figure 9.**
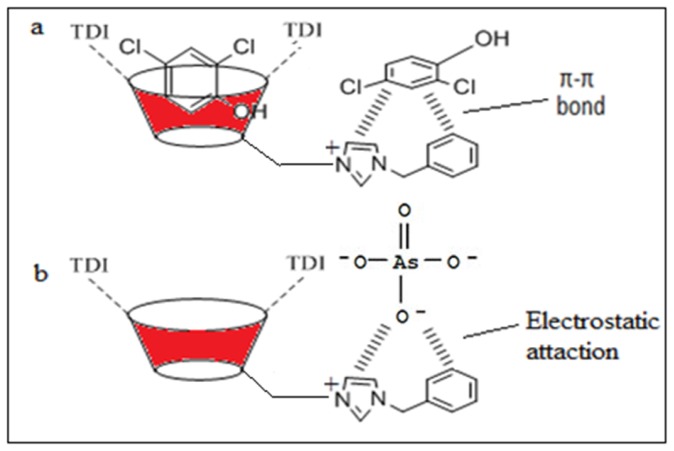
Schematic diagram on interaction between (**a**) βCD-BIMOTs-TDI polymer with phenols and (**b**) βCD-BIMOTs-TDI polymer with As(V).

Synthesized polymers are applied in sorption studies in order to compare the performance of βCD-BIMOTs-TDI polymer with native βCD-TDI polymer. The removal capacities of the polymers for phenols and As(V) in neutral condition are presented in Figure 8. It is found that the βCD-BIMOTs-TDI polymer enhances removal compared to the βCD-TDI polymer. The presence of the IL in βCD-BIMOTs-TDI polymer basically increases the selectivity towards phenol and As(V). Furthermore, it is well known that β-CD can form inclusion complex with phenols [37,38], so in this study inclusion complex could be formed as the cavity of β-CD was maintained in the polymerization process. Apart from that, the higher percentage of removal could be a result of the π–π interaction between the aromatic ring of phenols and imidazolium ring of βCD-BIMOTs-TDI polymer as shown in Figure 9a. The formation of the inclusion complex and π–π interaction was proven by using ^1^H NMR and 2D NOESY experiment in Section 2.9.

Moreover, the presence of imidazolium as a chelating group [39] in βCD-BIMOTs-TDI polymer able to remove As(V) from aqueous solution was due to the strong electrostatic interaction between the imidazolium ring and As(V), as shown in Figure 9b. Meanwhile, the presence of macropores also enabled us to apply it for removal studies since it reduces the diffusion distance for analyte molecules to transport, simultaneously increasing the adsorption and decreasing mass transfer resistance. Hence, the provision of a more favorable and fast adsorption process [30]. It can be speculated that βCD-BIMOTs-TDI polymer can interact well with phenols and As(V) because it possesses both the structural characteristics of benzylimidazolium and functional units of β-CD and it can be used in many areas for different applications.

### 2.10. Adsorption Behavior of βCD-BIMOTs via Inclusion Complex and π–π Interaction

**Figure 10.**
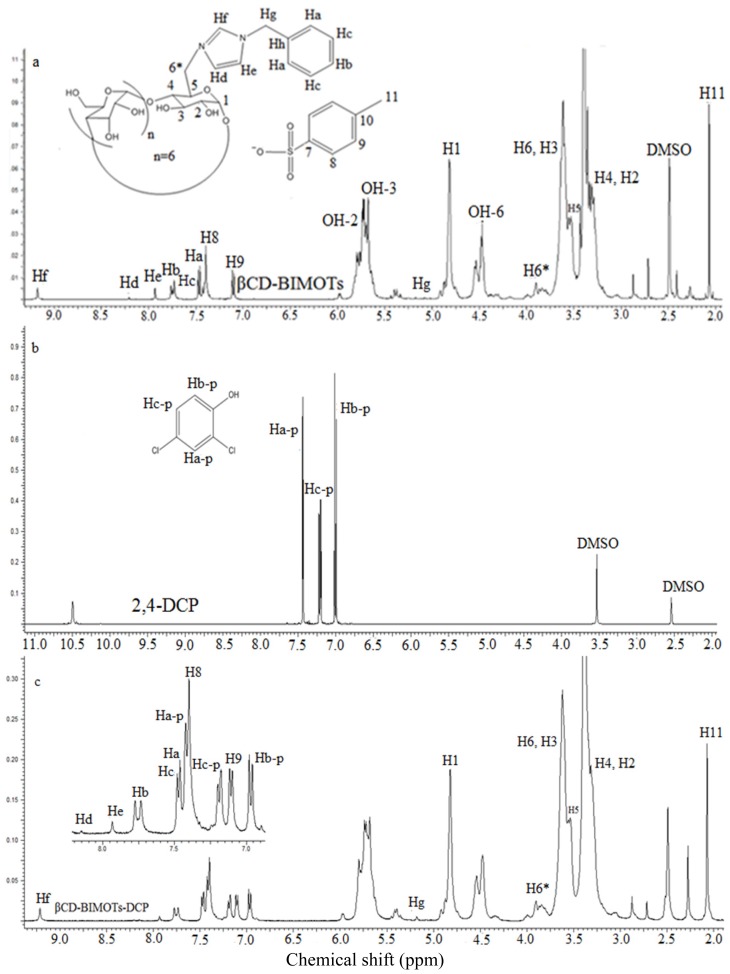
^1^H NMR spectrum of (**a**) βCD-BIMOTs; (**b**) 2,4-DCP and (**c**) βCD-BIMOTs-DCP.

**Figure 11.**
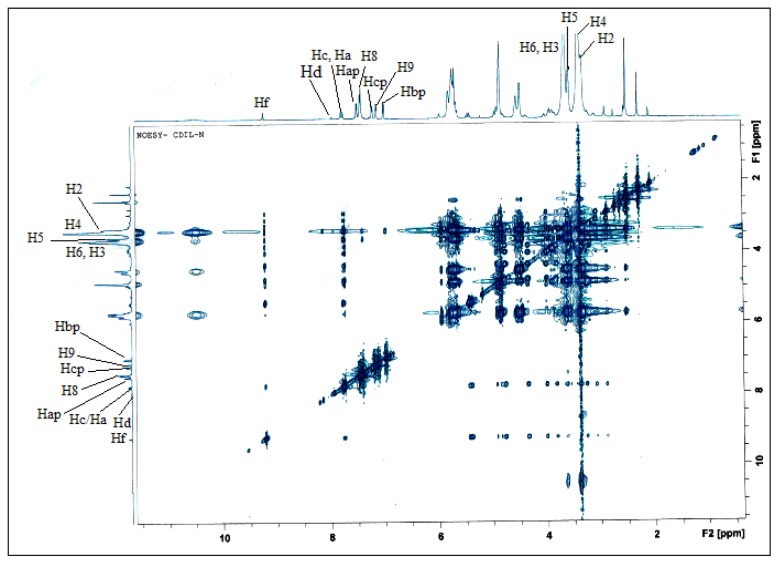
The two-dimensional NOESY spectrum of βCD-BIMOTs-DCP complex in DMSO-D_6_.

In order to prove the formation of inclusion complex and π–π interaction between modified β-CD (βCD-BIMOTs) with one selected phenol (2,4-DCP) ^1^H NMR and 2D NOESY experiment have been carried out. The analysis of the inclusion complex between modified β-CD (βCD-BIMOTs) and 2,4-DCP is crucial in this work, since the cavity of β-CD was maintained during the polymerization process. Furthermore, the findings have supported that inclusion complex formation is one of the main interactions between both the adsorbent and adsorbate in the adsorption process. In order to evaluate the geometry of inclusion formation of βCD-BIMOTs and 2,4-DCP, ^1^H NMR (Figure 10) and 2D NOESY measurements (Figure 11) (DMSO-D_6_, 25 °C, 600 MHz) were performed on a AVN600 spectrometer. The obvious downfield shift of the protons on the inner cavity of βCD-BIMOTs, *i.e*., H3 and H5 had been observed due to the anisotropic shielding by the ring current from the aromatic rings of 2,4-DCP (Table 3) compared to the other protons. Besides that, when 2,4-DCP enters into the hydrophobic cavity of βCD-BIMOTs, the change of the micro-environment in 2,4-DCP protons lead to the upfield shift (Hb-p and Hc-p). Meanwhile, H5 proton of βCD-BIMOTs changes from doublet to singlet upon the formation of inclusion complex as shown in Figure 10. The presence of proton signals belonging to both βCD-BIMOTs and 2,4-DCP molecules could be observed in the ^1^H NMR spectrum of 2,4-DCP-βCD-BIMOTs (Figure 10c) which strongly suggests that the new inclusion complex has formed.

The formation of inclusion complex was further proven by the 2D NOESY analysis (Figure 11) since 2D NMR is a powerful tool for investigating intermolecular interactions and for gaining more information on the conformation of the inclusion complex [40]. 2D NOESY experiments provide an upper limit (*ca.* 5Å) on the distance between protons cross peaks under favorable conditions. The cross-peaks in the spectra, indicated in Figure 11, originate from the interaction of the protons of 2,4-DCP and βCD-BIMOTs. The cross peaks of βCD-BIMOTs (3.5–3.6 ppm, H-3, H-5) and 2,4-DCP (7.4–6.9 ppm, Ha-p, Hb-p) demonstrate strong intensity. Hence, from the 2D NOESY spectra we can conclude that the aromatic ring of 2,4-DCP has been accommodated in the β-CD cavity. The cross peak around 7–8 ppm which belongs to BIMOTs and 2,4-DCP shows that there is an interaction between the BIMOTs ring and 2,4-DCP (Figure 11) and this could be due to both the π–π interaction. Therefore, we can conclude that higher percentage of removal between βCD-BIMOTs-TDI polymer and phenols could be due to inclusion complex formation and π–π interaction.

## 3. Experimental Section

### 3.1. Materials

β-CD is commercially available and was purchased from Acros (Acros, Geel, Belgium) (99%). 1-Benzylimidazole and toluene 2,4-diisocyanate (TDI) was supplied from Sigma Aldrich (Aldrich, Buches SG, Switzerland). Other reagents and chemicals were of the analytical reagent grade and were used and received without further purification. Double distilled water was used throughout the experiment. All the reactions were performed under inert conditions. *N*,*N*-Dimethylformamide (DMF) and hexane anhydrous were purchased form Merck (Merck, New York, NY, USA). *p*-Toluene sulfonic anhydride was prepared according to a literature procedure [22] and was used without further purification. Sodium arsenate dibasic heptahydrate (312 g/mol^−1^) was obtained from Sigma Aldrich (Aldrich, St. Louis, MO, USA). Arsenic (As)(V) and Phenols stock solution were prepared in double distilled water and their standards were prepared daily by having them diluted in water. The progress of the reactions were monitored by thin layer chromatography (TLC) using Merck TLC cards (70643) (Merck, New York, NY, USA) with butanol/ethanol (95%), water (5:4:3) as eluent for CD derivatives (β-CDOTs, βCD-BIMOTs) and the spots were visualized by using UV GL-58 Handheld UV-Lamp (UVP, Upland, CA, USA) or developed by dipping in 5% sulphuric acid (H_2_SO_4_) in ethanol followed by heating on a hot plate.

### 3.2. Characterization of the Samples

Fourier transform infrared (FT-IR) spectra were recorded on a Perkin–Elmer RX1 FT-IR (Perkin Elmer, Waltham, MA, USA) between 4000 and 400 cm^−1^ with a resolution of 2 cm^−1^. Samples were adequately mixed with KBr powder and pressed into disks. ^1^H NMR, ^13^C NMR and NOESY spectra were recorded on AVN 600 MHz (Bruker, Fällanden, Switzerland) and Dimethyl Sulfoxide (DMSO-D_6_) had been used as solvent. A sample of the polymers and monomer was dried in vacuum, and an elemental analysis of the sample was determined with a Perkin Elmer CHNS-2400 analyzer (Perkin Elmer, Waltham, MA, USA). The scanning electron microscope analysis for the morphology of the samples was obtained with a Leica S440 (Leica, Wetzlar, Germany). The Brunauer-Emmett-Teller (BET) analysis was determined from low-temperature nitrogen adsorption isotherms at 77.40 K using Quantachrome Autosorb Automated Gas Sorption System (Quantachrome, Boynton Beach, FL, USA). Typically, at least 1 g of sample (βCD-BIMOTs-TDI) was used each time during analysis. The surface area was obtained by the Brunauer–Emmett–Teller (BET) method, while average pore diameter and pore volume of βCD-BIMOTs-TDI in the dry state were measured from the adsorption branch of the isotherms by both the Barret–Joyner–Halenda (BJH) model (Quantachrome, Boynton Beach, FL, USA). X-ray diffraction (XRD) patterns were taken using Cu K_α_ irradiation with a Siemens D5000 X-ray diffractometer (voltage, 40 kV; current, 100 mA (Siemens, Frimley, UK). Powder samples were mounted on a sample holder and scanned from 5° to 30° at a speed of 3° per min. Thermo gravimetric analyses (TGA) curves were examined using a TA Instruments Q500 (Perkin Elmer, Waltham, MA, USA). A linear heating rate was set at 20 °C per minute within the temperature range from 50 to 900 °C in a stream of nitrogen atmosphere. Differential Scanning Calorimetry (DSC) (Perkin Elmer, Waltham, MA, USA) analysis was done by heating the samples from 30 to 400 °C at 20 °C per min.

### 3.3. Synthesis Method and Characterization

#### 3.3.1. Synthesis of 6-*O*-Monotosyl-6-deoxy-β-cyclodextrin (β-CDOTs) (1)

**Scheme 1:**
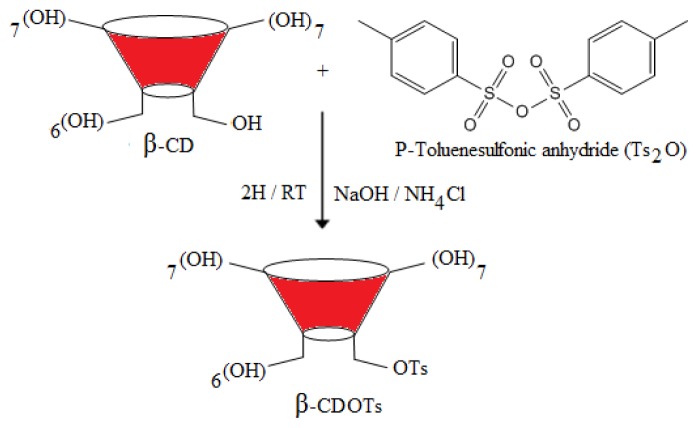
Synthesis of intermediate *O*-*p*-toluenesulfonyl-β-cyclodextrin (β-CDOTs).

A typical reticulation reaction would proceed as follows: tosyl-β-cyclodextrin (CDOTs) is prepared according to Zhong *et al*. [22] as shown in Scheme 1. OH_7_ in Scheme 1 represents 7 hydroxyl units at primary, secondary and tertiary position of β-CD while OH_6_ is the remaining hydroxyl group since IL reacts with only one hydroxyl group out of 7 units of β-CD. A suspension of β-CD (11.5 g, 10 mmol) and *p*-toluenesulfonic anhydride (Ts_2_O) (4.9 g, 15 mmol) in 250 mL of water is stirred at room temperature for 2 h. A solution of NaOH (5.0 g in 50 mL of H_2_O) is added, and after 10 min the reaction mixture is filtered through the celite on the sintered glass funnel to separate the non-reacting Ts_2_O. The filtrate was brought to pH-8 by the addition of ammonium chloride (13.4 g), affording **1** as a precipitate that is collected after cooling at 4 °C overnight and is used without further purification through chromatography. The degree of tolylation (DT) has been determined by using ^1^H NMR spectroscopy [41], which is based on the ratio of the areas of proton as shown in Equation (1);

(1) DT(%)=(AR/4)/([H1-H6]/7)×100

where *DT* (%) is the degree of tolylation, *AR* is the integral area of aromatic protons at § 7.8–7.4 ppm, and H1–H6 is the integral areas of the CD protons at § 5.9–3.2 ppm.

**IR/KBr**, cm^−1^ 3291 (OH), 2924 (C–H), 1646 (C=C), 1366 (SO_2_ Assym), 1153 (SO_2_ Sym). **^1^HNMR/ppm**, **DMSO-D_6_** H_8_ (7.74, d), H_9_ (7.43, d), OH_2_-OH_3_ (5.5–5.9, m), H_1_ (4,83, s), H_1_* (4.70, s), OH_6_ (4.2–4.6, m), H_3_,H_5_,H_6_ (3.2–3.60, m), H_2_–H_4_ (2.9–3.2, m), H_11_ (2.32, s). **^13^CNMR/PPM**, **DMSO-D_6_** C_7_ (144.7), C_10_ (132.6), C_9_ (129.8), C_8_ (127.5), C_1_ (101), C_4_ (81.6), C_2_ (73), C_3_ (72.7), C_5_ (72.4), C_6_ (59.9), C_11_ (21.1). CHNS (%) C (37.63), H (6.68), S (1.30). DT (%) = 0.83. Percentage yield (60%). Melting point (170 °C). TLC: *R*_f_ = 0.45.

#### 3.3.2. Synthesis of Mono-6-deoxy-6-(3-benzylimidazolium)-β-cyclodextrin (βCD-BIMOTs) (2)

**Scheme 2:**
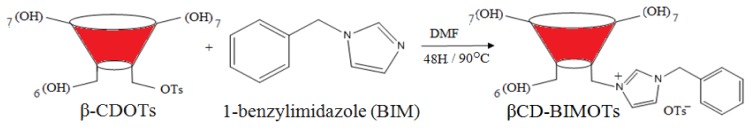
Preparation of monofunctionlized β-cyclodextrin (βCD-BIMOTs).

Furthermore, the reaction is observed, by reacting β-CDOTs with 1-benzylimidazole (BIM). Since tosyl is a good leaving group, imidazole can easily undergo nucleophilic substitution. The reaction is carried out in the DMF solvent since β-CDOTs and BIM forms a homogenous solution. The preparation of monofunctionlized β-cyclodextrin with BIM (βCD-BIMOTS) is done according to the following procedure [19] as shown in Scheme 2: Freshly dried CDOTs (1.00 g, 0.78 mmol) and appropriate amount of BIM (1.23 g, 7.8 mmol) in excess amount are dissolved in anhydrous DMF (40 mL) and the solution stirred at 90 °C in an inert atmosphere. After two days, the resultant solution is cooled to room temperature and slowly added into acetone. Then the mixture is stirred for 30 min and thereafter filtered and washed again with acetone. The product obtained is recrystallized three times from hot water to get the final product, **2** as a white yellow precipitate.

**IR/KBr**, cm^−1^ 3297 (OH), 2922 (C–H), 1652 (C=C), 1152 (C–N). **^1^H NMR/ppm**, **DMSO-D_6_** Hf (9.3, s), He (7.94, s), Hd (8.20,s),Hc (7.49, s), Hb (7.74, t), Ha (7.46,s), Hg (5.18,s),H_8_ (7.4, d), H_9_ (7.1, d), OH_2_–OH_3_ (5.5–5.9, m), H_1_ (4,81, s), OH_6_ (4.4–4.6, m), H6* (3.95), H_3_,H_5_,H_6_ (3.4–3.60), H_2_–H_4_ (3.2–3.4, m), H_11_ (2.07, s). **^13^C NMR/PPM**, **DMSO-D_6_** Ca (127), Cb (123.4), Cc (128.3), Cd (128), Ce (119), Cf (136.9), Cg (52), Ch (137.8), C_7_ (145.26), C_10_ (137.3), C_9_ (128.7), C_8_ (125.6), C_1_ (101.8), C_4_ (81.16), C_2_ (73.27), C_3_ (71.6), C_5_ (69.37), C_6_ (60.03), C_6_* (45.2),C_11_ (21.97). **CHNS (%)** C (38.2), H (6.67), S (0.47), N (1.0). Percentage yield (90%). Melting point (207 °C). TLC: *R*_f_ = 0.6.

#### 3.3.3. Synthesis of Insoluble β-Cyclodextrin Polymers

**Scheme 3:**
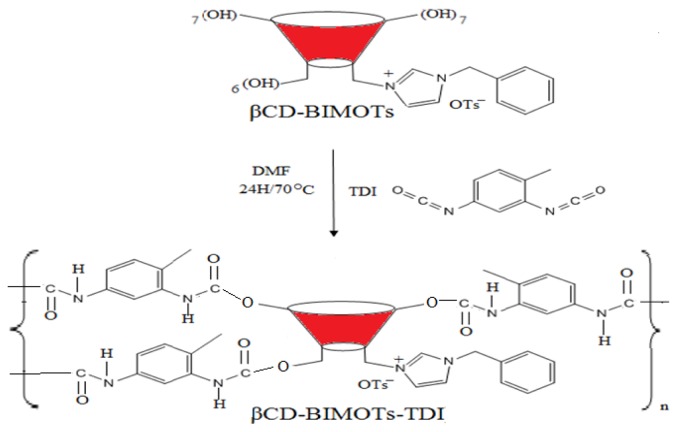
Synthesis pathway of β-cyclodextrin-ionic liquid polymer.

Insoluble β-cyclodextrin polymers (βCD-BIMOTs-TDI, βCD-TDI) are prepared according to the method of Mahlambi *et al.* [42] as shown in Scheme 3. The polymerization reaction is monitored using FT-IR spectroscopy. Briefly, 0.69 mmol of βCD-BIMOTs is first dissolved in 30 mL of anhydrous DMF at room temperature followed by of the addition of Toluene 2,4-diisocyanate (TDI) (6.9 mmol) dropwise and the mixture is stirred for 24 h at 70 °C. Meanwhile, the preparation of βCD-TDI is done as same as above procedure in 0.88 mmol of βCD and 8.8 mmol of TDI. The polymers formed are then precipitated with the addition of excess acetone. The solid formed are allowed to settle down in acetone for 10 min to allow for the removal of residual DMF from polymers followed by filtration and washed with acetone and double distilled water to remove the non-reactive cross-linker and dried overnight under reduced pressure. The dried polymer is first ground and sieved, using a 53 μm sieve, before being used.

### 3.4. Sorption Experiments

One of the potential applications of these polymers is that it can be used as an adsorbent for the removal of the micropollutants from the water (especially drinking water). Experiment data were determined by the following batch method: In each experiment 20 mg of dry polymer was mixed with 10 mL adsorbate at a known concentration in a tightly sealed flask. The solution was shaken for 2 h (phenols) and 17 h (As(V)) on a shaker at room temperature. The adsorbents were removed by filtration and the residual concentration was determined using Shimadzu (Kyoto, Japan) Ultraviolet-Visible spectroscopy (UV-vis) recording spectrophotometer equipped with 1 cm quartz cells for phenols, while 7500 series ICP-MS from Agilent Technologies (Palo Alto, CA, USA) was used to determine the concentration of As(V) in aqueous solution. The ICP-MS condition and setup information for As determination was shown in Table 4.

The percentage of adsorbate adsorbed on the polymer (removal efficiency, R (%)) was calculated using the following equation:

(2) $$R\%=(C_{\text{o}}-C_{\text{e}})/C_{\text{o}}\times 100$$

where, *C*_o_ and *C*_e_ are the initial and equilibrium concentration of solutions (mg/L), respectively.

## 4. Conclusions

A new CD-ILP (βCD-BIMOTs-TDI) has been successfully synthesized, characterized and compared with a native BCD-TDI polymer. The SEM result shows that the presence of IL increases the pore size while the TGA result shows that the presence of IL also increases the stability of the polymer. The BET result shows that surface area of the polymer decreases upon the functionalization with the IL and the Barret-Joyner-Halenda model reveals that it exhibits macropore size distribution, other than showing high sorption capacities towards phenols and As(V). A further study on the properties and its application is in progress.

## Supplementary Information



## Figures and Tables

**Table 1.** Main IR frequencies with assignments.

Samples	Wavelength (cm^−1^)	Assignments
βCD-TDI	3307	N–H and O–H stretch
	2270	Absence of isocyanate group
	1653, 1534	NHCO, carbamate linkage
	1450	Aromatic group in TDI
βCD-BIMOTs-TDI	3302	N–H, O–H stretch and imidazole ring
	2270	Absence of isocyanate group
	1600, 1412	Aromatic group in TDI
	1535, 1651	NHCO, carbamate linkage
	1153	C–N stretch

**Table 2.** Thermo gravimetric analysis result of samples.

Sample	Region (°C)	Weight loss (%)	Assignment
βCD-TDI	50–140	8	Water loss/Moisture
	260–365	68	Carbamate group and β-CD
	365–900	26	β-CD
βCD-BIMOTs-TDI	32–100	6	Water loss/Moisture
	270–357	46	Carbamate group, BIM, β-CD, OTs
	357–915	40	β-CD

**Table 3.** Chemical shift (δ) of βCD-BIMOTs, 2,4-DCP and βCD-BIMOTs-DCP.

Proton	βCD-BIMOTs	2,4-DCP	βCD-BIMOTs-DCP	Changes
	δ	δ	δ	Δδ
H1	4.8330		4.8345	+0.0015
H2	3.3030		3.3102	+0.0072
H3	3.5450		3.6231	+0.0781
H4	3.3268		3.3239	−0.0029
H5	3.3980		3.5567	+0.1587
H6	3.6298		3.6359	−0.0061
H8	7.4175		7.4138	−0.0037
H9	7.1269		7.1135	−0.0134
H11	2.0868		2.0834	+0.0034
Ha	7.4597		7.4829	+0.0232
Hb	7.7450		7.7454	+0.0004
Hc	7.4914		7.4957	+0.0043
Hd	8.2100		8.1502	−0.0598
He	7.9458		7.9480	+0.0026
Hf	9.2031		9.2226	+0.0195
Hg	5.1880		5.2032	+0.0152
Ha-p		7.4334	7.4300	−0.0034
Hb-p		7.0258	6.9765	−0.0493
Hc-p		7.2095	7.1135	−0.0960

**Table 4.** ICP-MS operating conditions for the ICP-MS equipped with an octopole reaction system.

Parameter	Value
RF Power	1550 watts
RF Matching	1.55 V
Reflected Power	0 W
Sample Uptake Time	30 sec
Sample Uptake Rate	0.4 r sec^−1^
Chamber temperature	2 °C
Nebuliser	Babington
Cones	Ni
Coolant Argon Flow Rate	15 L min^−1^
Carrier Gas Flow Rate	1.2 L min^−1^
Auxiliary gas flow rate	0.9 L min^−1^
Water RF/TP Flow Rate	2.4 L min^−1^
Water RF/TP Temperature	20 °C
Mode	He
Internal standard	^72^Ge
Integration time (sec per point)	1
