# Supplementary material for: Synthesis and Characterization of β-Cyclodextrin Functionalized Ionic Liquid Polymer as a Macroporous Material for the Removal of Phenols and As(V)
Source: Int J Mol Sci. 2013 Dec 23;15(1):100–19. doi: 10.3390/ijms15010100 (PMC3907800; doi:10.3390/ijms15010100)
Supplement: Supplementary file 1 [file ijms-15-00100-s001.pdf]

Supplementary Information

Table S1. Structural parameters of the samples.

Polymers	Surface area (m^2/g)	Pore volume (cm^3/g)	Pore diameter (nm)	Pore size distribution
βCD-TDI	2.401	0.02188	1.585	<2 nm (micro pore)
βCD-BIMOTs-TDI	1.254	0.02435	77.66	>50 nm (macro pore)

Table S2. DSC analysis of the samples.

Samples	Temperature (℃)	Peak (℃)	Exo/Endo	Assignment
βCD-TDI	38–93	60	Endothermic	Loss of water
	280–343	323	Endothermic	Melting of β-CD and TDI
	343–350	350	Endothermic	Melting of β-CD
	350–357	356	Exothermic	Curing process
βCD-BIMOTs-TDI	36–79	70	Endothermic	Loss of water
	252–343	330	Endothermic	Melting of β-CD, TDI, BIMOTs

© 2013 by the authors; licensee MDPI, Basel, Switzerland. This article is an open access article distributed under the terms and conditions of the Creative Commons Attribution license (http://creativecommons.org/licenses/by/3.0/).
